# Contrast-Enhanced Ultrasound (CEUS) Evaluation of Canine Prostatic Hyperplasia before and after Osaterone Acetate Therapy: Preliminary Results

**DOI:** 10.3390/ani14111683

**Published:** 2024-06-05

**Authors:** Giorgia Pettina, Roberta Bucci, Antonio Mazzetti, Marco Quartuccio, Domenico Robbe, Maria Carmela Pisu

**Affiliations:** 1 Department of Veterinary Science, University of Messina, Viale Palatucci 13, 98168 Messina, Italy; giorgia.pettina@studenti.unime.it (G.P.); marco.quartuccio@unime.it (M.Q.); 2 Department of Veterinary Medicine, University of Teramo, Loc. Piano d’Accio, 64100 Teramo, Italy; mazzettiantonio7@gmail.com (A.M.); drobbe@unite.it (D.R.); 3 VRC—Centro di Referenza Veterinario, Corso Francia, 10138 Torino, Italy; mariacarmela.pisu@vierreci.it

**Keywords:** canine prostatic disease, ultrasound imaging, benign prostatic hyperplasia, CEUS, osaterone acetate

## Abstract

**Simple Summary:**

The contrast-enhanced ultrasound (CEUS) allows the evaluation of the vascularization of an organ, using contrast agents composed of gas microbubbles, such as sulfur hexafluoride. Prostate perfusion is increased in dogs with benign prostatic hyperplasia (BPH), due to the stimulation of dihydrotestosterone (DHT). In the present study, CEUS is applied for monitoring dogs with BPH, receiving osaterone acetate (OSA). Fifteen intact adult dogs are referred for BPH. A CPSE assay and B-mode ultrasound confirm the diagnosis. CEUS is performed before OSA treatment (D_0_) and highlights the rapid diffusion times of the contrast agent (length of the wash-in and wash-out phases). Prostatic cysts and parenchymal alteration are also detected. After treatment (D_1_), the CPSE and prostate volume are significantly reduced, while CEUS detects a significant increase in wash-in and wash-out times and a reduction in parenchymal abnormalities. These findings confirm the decrease in prostatic perfusion after OSA treatment and, although preliminary, are promising for the broader use of CEUS for the diagnosis and monitoring of dogs with BPH.

**Abstract:**

The prostate is the only sexual gland of the male dog, and dihydrotestosterone (DHT) regulates its growth. In intact dogs, constant DHT stimulation results in benign prostatic hyperplasia (BPH) that can be treated with osaterone acetate (OSA). This study describes the effects of OSA treatment, detected by contrast-enhanced ultrasonography (CEUS), highlighting prostatic vascularization with a contrast agent composed of gas microbubbles. Fifteen dogs (2–8 years) of different sizes and breeds (4–30 kg) diagnosed with BPH are involved in the study. Before treatment (D_0_), CPSE is measured (294.05 ± 115.97 ng/mL), and a B-mode ultrasound is performed (V*ratio* = 2.80 ± 1.85), confirming BPH. CEUS highlights the length of the wash-in (11.93 ± 2.08 s) and wash-out (42.20 ± 6.99 s) phases of the contrast agent in the prostate and the presence of cysts and parenchymal alteration. Dogs are treated with OSA (0.5 mg/kg for 7 days) and reassessed after 21 days (D_1_): CPSE and prostate volume are significantly (*p* < 0.001) reduced. The length of the wash-in (14.73 ± 2.54 s) and wash-out (51.13 ± 6.03 s) phases are significantly (*p* < 0.001) increased. The results confirm the effectiveness of the treatment, particularly the reduction in prostatic perfusion, confirmed by the increase in diffusion times of the contrast. Although preliminary, these findings are promising for the use of CEUS in monitoring dogs with BPH.

## 1. Introduction

The prostate is the sole male accessory sex gland in the canine species [1]. This androgen-dependent organ, with its secretion, contributes to the main volume of the canine ejaculate [2]. In dogs, the prostatic gland is typically a pelvic or abdominal organ, depending on its size, bladder repletion, and animal age, as it surrounds the proximal urethra and the neck of the urinary bladder [3,4]. The prostatic fluid, or seminal plasma, is clear and rich in zinc, provides an adequate environment for sperm and seems to have an antibacterial function [5,6]. The gland’s activity is regulated by a biologically active metabolite of testosterone, the dihydrotestosterone (DHT), produced in the prostatic epithelial cells by the enzyme 5α-reductase [6]. As the dog ages, the activity of 5α-reductase seems to increase, resulting in the greater production of DHT [7], thus determining the onset of one of the most widespread prostatic pathologies in the canine species, benign prostatic hyperplasia (BPH) [8]. Other less frequent prostatic conditions are prostatitis, prostatic cysts, abscesses, squamous metaplasia, and neoplasia (mainly adenocarcinomas and transitional cell carcinomas) [1,8].

BPH is an age-related disorder that is more likely to occur in intact male dogs that have exceeded 40% of their expected longevity [9,10], characterized by a non-cancerous increase in the size of the gland, mainly determined by prostatic cell hyperplasia and, partly, by hypertrophy [1,3,7]. Clinical signs are frequently mild to absent [1]; however, affected dogs may suffer from subfertility due to an alteration in the quality of seminal plasma. It is also possible to highlight blood contamination of the first and third fractions of the ejaculate of prostatic origin [7]. Other clinical signs may include hematuria, sero-hemorragic preputial discharge, and tenesmus [6]. In fact, as, in the canine species, prostate growth is predominantly eccentric in a dorsal direction [7], this causes the compression of the rectum, and, consequently, constipation, dyschezia, and, sometimes, perineal hernias. An early diagnosis helps preserve the health and fertility of affected dogs. To detect BPH, an ultrasound (US) exam can be performed to measure the actual volume of the prostate and correlate it with the expected volume. A ratio between the two measurements greater than 1.5 indicates an increase in prostate volume [9,11]. Canine Prostatic Specific Esterase (CPSE) is a useful marker of prostatic activity. This is a major prostatic secretion, representing 90% of prostatic fluid proteins [7], is influenced by DHT, and can also be measured in the blood [12]. The serum CPSE concentration increases nonspecifically in the case of prostatic disease, and a preventive threshold value of 52.3 ng/mL has been identified [11]. Values above 60 ng/mL have been correlated with an increase in prostate volume, without clinical signs; values higher than 90 ng/mL correlate with a more than doubled prostate volume [9]. However, serum CPSE also increases following ejaculation, so, for a reliable measurement, dogs must be at sexual rest for at least 24 h [13]. Regarding treatment, the goal should be to remove the androgen stimulation of the prostate permanently or provisionally. This can be achieved definitively by carrying out an orchiectomy or, temporarily, by using molecules that prevent the action of DHT on the prostate. It is possible to use 5α-reductase inhibitors (such as finasteride), which reduce the conversion of testosterone into its active form. Currently, however, the only registered drug to treat BPH in the canine species is osaterone acetate, a steroidal androgen that acts by both binding to prostatic androgen receptors and inhibiting the action of 5α-reductase [1,14,15]. Treatment with 0.25–0.5 mg/kg of osaterone acetate is reported to reduce prostate size for up to 6 months [16]. However, a recent study reports a transient adverse effect on epididymal function and sperm maturation, with an increase in tail defects, which resolves at 4 months post-treatment [17]. Furthermore, GnRH agonists (such as deslorelin acetate), that inhibit the production of luteinizing hormone (LH), can be used, in association with osaterone acetate, thus temporarily interrupting the production of testosterone by the Leydig cells [1], in dogs not intended for reproduction.

For prostate evaluation, the B-mode ultrasound is certainly the technique of choice, allowing the evaluation of both the dimensions and the characteristics of the parenchyma [10,18]. In addition to the B-mode exam, further ultrasound methods are available for increasingly detailed and early information, such as the Doppler exam, which allows the evaluation of prostatic vascularization and blood flow [19], and, recently, also the strain and 2D-shear wave elastography technique, described for the study of the prostatic tissue elasticity [20]. Recently, the contrast-enhanced ultrasound (CEUS) has become an increasingly useful tool in veterinary medicine to identify organ perfusion and vascularization [21]. The CEUS technique is based on the intravenous injection of a contrast agent consisting of gas microbubbles less than 7 microns in size. Unlike the agents used for CT and MRI, gas microbubbles do not diffuse into the extracellular space, and, being smaller than red blood cells, their use does not cause an embolism [21]. The injection of contrast agents determines a transient enhancement in the echo signal which can be detected with contrast harmonic software. The kinetics of vascularization can thus be evaluated quantitatively by interpreting the increase in signal intensity from the arrival of the contrast agent (wash-in) to its elimination (wash-out) [22]. A widely used contrast agent is composed of microbubbles filled with sulfur hexafluoride, characterized by prolonged stability and the uniformity of bubble dimensions, which improves their harmonic behavior [23].

The first applications of CEUS in veterinary medicine involved the liver, kidneys, spleen, and lymph nodes [22,24]. Recently, this technique has also found wide application in canine reproduction [25,26,27]. The application of CEUS for the evaluation of ovarian activity [28] and pregnancy [29,30], but also during pyometra [31,32], has been described in the bitch. For male dogs, there are several studies on both healthy [33] and pathological testes [34,35,36]; furthermore, some studies have been published on the prostate in healthy [37,38,39], neutered [40], and pathological animals [22,37,41,42,43].

A limitation in the imaging of canine prostatic diseases is that they are slightly differentiable in B-mode or Doppler examinations [41,43]. In this context, the CEUS technique, compared to Doppler, allows organ perfusion to be detected even in small-caliber vessels [22,40]. Furthermore, CEUS also allows quantifying perfusion by measuring transit times in the tissue (wash-in and wash-out of the contrast agent). These parameters are mainly valuable for the diagnosis of prostate tumors, which show variable transit times [43]. Differently from other prostatic pathologies, tumors respond poorly to anti-androgen treatments and there are often risks of dissemination which limit the use of biopsies or fine-needle aspiration [1,3]. In the authors’ opinion, deepening the ultrasound study of prostatic pathologies, also in response to therapies, using new techniques such as CEUS, is relevant in order to identify early and treat pathological alterations.

Although several studies have been published investigating prostatic vascularization in dogs suffering from BPH [18,44] and its variation following therapy [15,45,46], to the authors’ knowledge, a CEUS evaluation has never been described in animals undergoing treatment with OSA. This manuscript aims to describe the preliminary findings obtained with the CEUS technique, using a hexafluoride-based contrast agent, in patients affected by BPH before and after treatment with osaterone acetate.

## 2. Materials and Methods

### 2.1. Animals

The current clinical study was performed in 2023 on owned adult intact male dogs, referred to VRC—Centro Di Referenza Veterinario, in Turin, for andrological evaluation and diagnosed with BPH. Fifteen dogs aged 2 to 8 years of different body weights (BW) (4–30 kg) and breeds were enrolled (Table 1). The same team of adequately trained veterinarians performed all procedures.

Each animal underwent a general clinical examination, serum chemistry (BUN, creatinine, ALP, and ALT; total proteins, albumin, and electrolytes), complete blood cell count, serum CPSE assessment, and prostate ultrasonography on the day of diagnosis (D_0_).

Patients who showed clinical signs, ultrasound findings, and CPSE values (over 53 ng/mL) compatible with BPH underwent therapeutic treatment with 0.5 mg/kg of osaterone acetate (Ypozane^®^, Virbac, Milano, Italy) (OSA), administered orally for seven consecutive days. Serum CPSE level was also dosed 21 days after OSA treatment (D_1_).

### 2.2. B-Mode and Contrast-Enhanced Ultrasound Procedures

Ultrasound procedures were performed on each animal on the day of diagnosis (D_0_) and after 21 days from OSA treatment (D_1_) using a veterinary scanner (My Lab X8 VET, Esaote, Genova, Italy) equipped with a 7.5 MHz linear, and a microconvex transducer. Dogs were positioned in dorsal or lateral recumbency, hair was clipped when necessary, and acoustic gel was applied directly to the transducer; no sedation was required for the procedure. The urinary bladder was filled with a moderate amount of urine to move the prostate cranially and to reduce acoustic shadowing caused by the pelvic bone.

B-mode ultrasound (US) examination allowed the evaluation of prostatic echotexture, highlighting the presence of any cystic or nodular lesions and the size of the gland to be estimated. The prostate was scanned in longitudinal and sagittal planes to assess prostatic height (H), length (L), and width (W). For each animal, the V*ratio* [11] was also calculated according to the following formulae [11,47,48]:

Actual Volume (Va) = H × W × L × 0.523

Expected Volume (Ve) = (0.33 × BW) + 3.28

V*ratio* = Va/Ve

**Figure 1:**
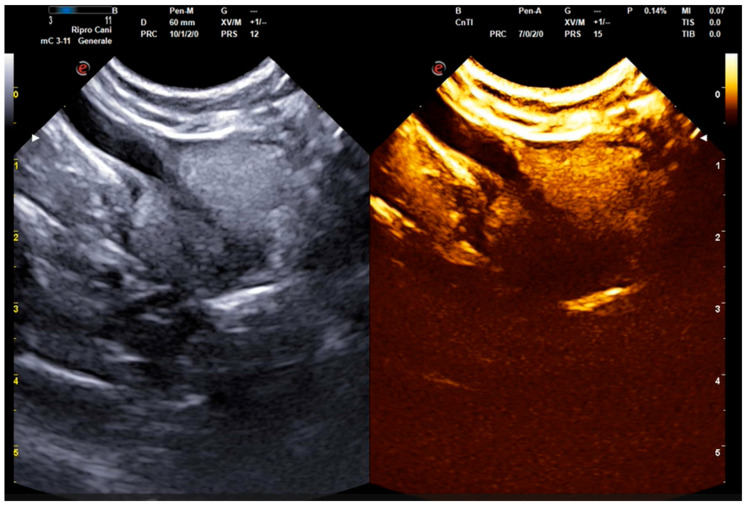
Dual-live function displaying simultaneously B-mode and contrast-enhanced images (My Lab X8 VET, Esaote, Genova, Italy). Detail of the pre-treatment (D_0_) wash-in phase in a 4-year-old Mixed Breed.

For contrast-enhanced ultrasonography (CEUS), a sulfur hexafluoride contrast agent was used (SonoVue^®^, Bracco Imaging, Milan, Italy). Briefly, the agent was reconstituted, following the manufacturer’s recommendations, by diluting the contrast agent powder with 5 mL of sterile saline and shaking vigorously for 20 s. All animals received an intravenous dose of 0.03–0.04 mL/kg, followed by a bolus of 5 mL of sterile saline, to wash in the contrast agent [22,31]. The machine allowed a dual-live function with B-mode and contrast images displayed simultaneously (Figure 1). For all patients, the length of the wash-in and wash-out phases of the contrast agent in the prostate were assessed (expressed in seconds) using a timer activated simultaneously with contrast inoculation [35]. The wash-in phase was calculated starting from the arrival of the contrast at the gland until reaching the peak intensity (time to reach peak intensity—TTP) [41]; the wash-out phase was considered the period from TTP until the contrast was eliminated.

### 2.3. Statistical Analysis

Data obtained were analyzed using the JASP program (JASP, version 0.17, computer software, University of Amsterdam, Amsterdam, The Netherlands). The Shapiro–Wilks test was used to assess data normality. Normally distributed data underwent a descriptive study (mean, standard deviation, and range). Serum CPSE levels, B-mode, and contrast-enhanced ultrasonographic findings performed pre- (D_0_) and post- (D_1_) treatment were compared with Student’s *t*-test (*p* < 0.001) to highlight statistically significant differences.

## 3. Results

All animals were in good general clinical condition, and blood test parameters did not show significant alterations.

Before treatment (D_0_), clinical and laboratory findings were consistent with the diagnosis of BPH: mild (hematuria, tenesmus, and sanguineous discharge from urethra) to absent clinical signs were reported in all dogs; the serum CPSE levels were above the cut-off value (53.2 ng/mL [11]) for all dogs, ranging from 70 to over than 500 ng/mL. The B-mode US highlighted an increase in the size of the prostate in all subjects examined, with an average actual volume (Va) [47] of 26.16 ± 22.25 cm^3^. Consequently, in all cases under examination, the V*ratio* was higher than the limit of 1.5 [9] (range 1.15–7.49). Regarding the echotexture, cystic prostatic degeneration was also highlighted in five animals.

After OSA treatment (D_1_), an improvement in clinical and laboratory findings was highlighted for all patients. No clinical signs were reported, and the serum CPSE level was significantly (*p* < 0.001) reduced compared to pre-treatment values, showing average values of 217.31 ± 76.12 ng/mL. Prostatic cysts, identified in five dogs, reduced in volume after treatment. The height (H), length (L), and width (W) of the prostate were also significantly reduced compared to pre-treatment values. The actual volume (Va) after treatment was, on average, 7.18 ± 9.19 cm^3^, while the V*ratio* was between 0.09 and 2.85. The Student *t*-test highlighted a significant (*p* < 0.001) difference between pre- and post-treatment values for all data considered. Table 2 summarizes the average values obtained.

The study performed with the CEUS technique allowed a qualitative evaluation of the prostate perfusion rate before (D_0_) and after (D_1_) treatment with OSA. The data obtained highlighted that, after medical therapy, prostatic perfusion was reduced. The wash-in and wash-out phases were significantly (*p* < 0.001) longer on D_1_ compared to D_0_ (Table 3).

**Figure 2:**
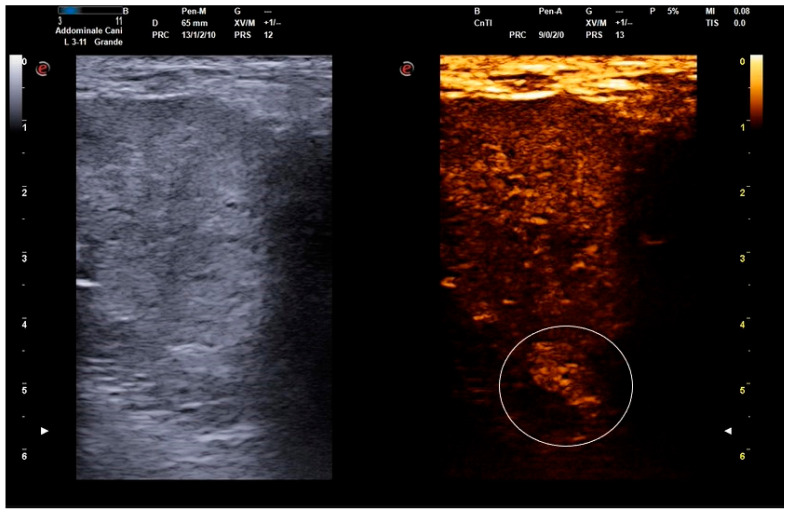
Dual-live function displaying simultaneously B-mode and contrast-enhanced images (My Lab X8 VET, Esaote, Genova, Italy). Detail of the pre-treatment (D_0_) wash-in phase in an 8-year-old Dachshund. The B-mode image highlights the presence of prostatic cysts. The contrast-enhanced image also highlights the presence of a parenchymal alteration (white circle), not clearly visible with the B-mode function. Cystic lesions are also evident in the contrast-enhanced image, resulting in a heterogeneous appearance.

Furthermore, the CEUS technique highlighted, in three dogs, the presence of parenchymal alteration, not highlighted in B-mode, the size of which was reduced following OSA therapy. Even cystic lesions were highlighted by contrast, which delimitated the cavities. This gave a heterogeneous appearance to the prostatic parenchyma, even in the contrast-enhanced image (Figure 2).

## 4. Discussion

BPH is a common pathology in intact male dogs that can be asymptomatic in the initial stages but progressively leads to the appearance of clinical signs, such as hematuria, tenesmus, sanguineous discharge from the urethra, and ribbon-like stools [49]. The literature on this pathology highlights how BPH occurs, on average, in dogs over 5 years but is also linked with the animal size [2]. Therefore, it is important to have regular prostate checks in all intact dogs that exceed 40% of their expected longevity [9,10]. It is interesting to note how, in the present study, since a group of dogs of different sizes and ages was used, the average age of the group was 5 years, in line with what is reported in the literature. Furthermore, in all animals included, clinical signs were mild to absent, despite an established prostatic hyperplasia, as they were regularly screened, and, therefore, the condition was diagnosed early. A BPH diagnosis was confirmed with the B-mode US exam: in all dogs included in the study, the ratio (V*ratio*) between the actual (Va) and expected (Ve) volume was, on average, higher than 2.5, and, therefore, highly indicative of prostatic hyperplasia, as reported by Holst et al. [9]. The same author reports that, in subjects affected by BPH, CPSE values > 90 ng/mL correlate to a V*ratio* > 2.5 [9,50]. In the cases under examination, the CPSE values were, on average, higher than the previously reported threshold value, with a range of 70–500 ng/mL. The co-presence of CPSE > 90 ng/mL and V*ratio* > 2.5 allowed the authors to confirm the diagnosis of BPH for all patients. No biopsy or fine-needle aspiration (FNA) was performed to confirm BPH, as clinical signs and ultrasound findings are reliable for a definitive diagnosis, as suggested in a study by Rodak et al. [49]. Furthermore, the present clinical study was conducted on owned animals; therefore, only the procedures necessary to obtain a diagnosis and follow-up were performed, while moderately invasive exams, such as biopsy or FNA, were excluded from the experimental design, as not strictly necessary. As for the formulae used to estimate prostate volume, several equations have been published over the years. Various authors [51,52] proposed equations for estimating the real volume obtained using linear regression. The formula by Kamolpatana et al. [52] was also used by Holst et al. [9] for their study on V*ratio*. However, the authors chose to use the formula suggested by Alonge et al. [11], estimating the real volume of the prostate using the ellipsoid formula, as also recommended by Ruel et al. [47] in a study on the correlation between prostate volume and body weight. Various formulae are also available to estimate the expected volume, starting from body weight [48,53], derived from studies on cadavers. The formula by Sannamwong et al. [48] is also reported for the V*ratio* studies [9,11]; therefore, it was also used in the current study.

The post-treatment clinical and ultrasound checks were carried out 21 days (D_1_) after OSA administration, since, on that date, it is possible to highlight the reduction in both clinical signs and prostate volume. A series of papers by Nizanski et al. [15,54] highlighted that using osaterone acetate determines a reduction in clinical symptoms and an improvement in ultrasound findings after 7–14 days from the start of therapy. Osaterone determines a faster clinical improvement than other drugs, such as deslorelin implants [15,54], and is more manageable and requires a shorter administration protocol than other molecules, such as finasteride [1]. It can also be used in stud dogs, as it does not block spermatogenesis, unlike GnRH analogs. The animals included in the study were then treated with OSA, as it is a safe and effective drug for treating BPH. Moreover, it is the only drug registered in Italy for the treatment of prostatic hyperplasia in dogs. The statistical study performed on the data obtained confirmed that, after 21 days, treatment with OSA significantly reduced prostate volume and serum CPSE levels. A significant reduction in CPSE after OSA treatment is also described in a recent review by Alonge et al. [55], reporting the data obtained on a group of animals with subclinical BPH. It is, instead, interesting to note that, in a study on the treatment of BPH with finasteride [56], the reduction in CPSE 28 days after the start of treatment was not statistically significant but was still noticeable. Compared to threshold values, the CPSE values remained elevated after treatment with OSA, although a significant reduction was highlighted (*p* < 0.001) compared to pre-treatment values. This is justified by having performed a control analysis 21 days after therapy. In fact, in the authors’ experience, more time is needed for the CPSE to return to values of around 50 ng/mL, especially in subjects with values over 200 ng/mL, as in the animals involved.

It also reduced the perfusion of the gland, resulting in a significant increase in the wash-in and wash-out phases, highlighted with CEUS. Several studies in the literature [7,39,45] highlight an increase in prostate perfusion in dogs affected by BPH, characterized, upon examination with Power Doppler (PW), by an increase in peak systolic velocity peak diastolic velocity, and maximum average velocity [7]. A limitation of the present research is the lack of a comparison between CEUS and PW regarding pre- and post-treatment perfusion. However, a study by Polisca et al. [57] on the use of deslorelin acetate in dogs with BPH highlights a reduction in blood flow, which can be demonstrated with a PW examination approximately 40 days after implant application. According to the authors, this reduction is due to the decrease in testosterone levels, which also acts on the prostatic blood supply, as well as on the size of the organ. The reduction in prostatic size and blood supply can be seen earlier in the present study, already 21 days after treatment, as OSA acts directly on testosterone, preventing its conversion into DHT, while deslorelin acts by determining a downregulation of the hypothalamus–pituitary–gonadal axis [15]. Another recent paper by D’Francisco et al. [46] highlighted, using Doppler US, a reduction in blood flow anomalies and an increase in the arterial resistivity index in patients with BPH 30 days after an experimental treatment with acyline, a third-generation GnRH antagonist.

As for CEUS, Russo et al. [38] published the first study describing prostatic perfusion and highlighted how, in healthy sedated dogs, contrast inoculation highlights a homogeneous enhancement of the gland approximately 15 s after its administration. Even the wash-out phase is homogeneous. Troisi et al. [22] support these results, describing the use of CEUS in patients with BPH. In this case, the wash-in phase is characterized by a chaotic appearance of the vascularization, which also highlights the cystic lesions of the gland. The results obtained in the present study confirm those findings: in subjects with cystic lesions, the contrast-enhanced image is characterized by a heterogeneous appearance. Furthermore, the data obtained showed how CEUS highlights parenchymal alteration not clearly distinguishable on the B-mode examination. These data, also in agreement with Troisi et al. [22], could be valuable in clinical practice to identify more precisely the areas of the parenchyma to be subjected to sampling with FNA or biopsy. Further studies are planned in this regard. Regarding the wash-in and wash-out times, a study by Vignoli et al. [41] presents the time, expressed in seconds, to reach the maximum intensity peak (TTP) in the contrast-enhanced images. The first difference between the reference and the present study is the moment when time begins to be measured. In the cited paper, the starting moment to calculate the TTP is identified as the injection of the contrast medium, and the TTP in healthy animals is 33.6 ± 6.4 s, while, in dogs with BPH, it is 26.2 ± 5.8 s. Similar results are also reported by Troisi et al. [22], describing TTPs of 39.38 s and 26.27 s for healthy and pathological male dogs. In the present study, however, although the procedure was timed starting from the injection of the contrast, the wash-in phase was measured from the arrival of the contrast agent in the organ until the peak intensity was reached; the wash-out, not reported in the cited papers, was calculated from the peak until the contrast was eliminated from the prostate. This causes a discrepancy in the data obtained. Before the treatment with OSA (D_0_), a wash-in phase of 11.93 ± 2.08 s was recorded, much lower than the value reported by [22,41] for patients with BPH. This difference, however, is justified by the findings of Russo et al. [38], who described the arrival of the contrast agent at the prostate about 15 s after administration. If this time interval is considered, the data presented for the wash-in length agree with what is reported in the literature [38,41]. To the authors’ knowledge, there are no studies on the length of the wash-out phase in the prostate of healthy or pathological animals. However, in the present study, after treatment, the wash-out phase was significantly longer than pre-treatment values, confirming the reduction in blood flow following OSA therapy.

## 5. Conclusions

Benign prostatic hyperplasia is a frequent disease in adult and elderly dogs, which can also be managed using drugs such as osaterone acetate. The data obtained in the present paper highlight how B-mode US and CEUS can provide valuable information for diagnosis and monitoring therapy for BPH. To the authors’ knowledge, this is the first study where CEUS is applied for monitoring OSA therapy for BPH. Although the study was conducted on a limited number of animals, the results are encouraging. The application of this advanced imaging method can provide qualitative data (wash-in and wash-out phase times) useful for BPH follow-up after treatment. Further studies are required to validate these preliminary results obtained, also including biopsy and/or FNA to confirm the BPH diagnosis. Although the CEUS technique requires adequately trained personnel and specific equipment, the preliminary results of the study are promising for the broader use of the CEUS technique in clinical practice for dogs with prostatic diseases such as BPH.

## Figures and Tables

**Table 1:** Breed, body weight (BW), and age of dogs enrolled in the study.

Dog	Breed	BW (kg)	Age (Years)
1	Dachshund	12	8
2	Yorkshire Terrier	5	5
3	Australian Shepherd	24	5
4	Yorkshire Terrier	6	6
5	Fonni Shepherd	30	6
6	Dachshund	10	7
7	Dachshund	4.3	3
8	English Bulldog	20	4
9	Mixed Breed	26.6	7
10	English Bulldog	24	3
11	Boston Terrier	9	6
12	Australian Shepherd	25	4
13	American Pitbull Terrier	26	2
14	Mixed Breed	18	4
15	Mixed Breed	22	5

**Table 2:** Average values (expressed as mean ± standard deviation and range) of the prostatic measurements (height, H; length, L; and width, W) obtained with ultrasound, the actual volume (Va), the expected volume (Ve), the V*ratio* (Va/Ve), and the CPSE, recorded before (D_0_) and after 21 days (D_1_) from treatment with osaterone acetate. Significance was assessed with the Student *t*-test (*p* < 0.001).

	D_0_	D_1_	*p*
H (cm)	3.58 ± 0.49 (2.7–4-3)	2.12 ± 0.81 (1.3–4.5)	<0.001
L (cm)	3.47 ± 0.85 (2.2–4.82)	2.32 ± 0.62 (1.5–3.2)	<0.001
W (cm)	3.54 ± 1.60 (1.7–6.4)	1.98 ± 1.17 (1–4.5)	<0.001
Va (cm^3^)	26.16 ± 22.25 (9.94–69.08)	7.18 ± 9.19 (1.09–33.89)	<0.001
Ve (cm^3^)	9.04 ± 2.92 (4.69–13.18)	9.04 ± 2.92 (4.69–13.18)	/
V*ratio*	2.80 ± 1.85 (1.15–7.49)	0.72 ± 0.75 (0.09–2.85)	<0.001
CPSE ng/ml	294.05 ± 115.97 (70–500)	217.31 ± 76.12 (70–380)	<0.001

**Table 3:** Average values (expressed as mean ± standard deviation and range) of the wash-in and wash-out phases, recorded before (D_0_) and after 21 days (D_1_) from treatment with osaterone Acetate. Significance was assessed with the Student *t*-test (*p* < 0.001).

	D_0_	D_1_	*p*
Wash-in (s)	11.93 ± 2.08 (8–16)	14.73 ± 2.54 (10–21)	<0.001
Wash-out (s)	42.20 ± 6.99 (35–60)	51.13 ± 6.03 (46–65)	<0.001

## Data Availability

The data presented in this study are available on request from the corresponding author.
